# Targeting the Endocannabinoid System for Neuroprotection: A ^19^F-NMR Study of a Selective FAAH Inhibitor Binding with an Anandamide Carrier Protein, HSA

**Published:** 2013

**Authors:** Jianqin Zhuang, De-Ping Yang, Xiaoyu Tian, Spyros P. Nikas, Rishi Sharma, Jason Jianxin Guo, Alexandros Makriyannis

**Affiliations:** 1 Center for Drug Discovery, Department of Pharmaceutical Sciences, and Department of Chemistry and Chemical Biology, Northeastern University, 360 Huntington Avenue, Boston, MA 02115, USA; 2 Department of Chemistry, The College of Staten Island, City University of New York, 2800 Victory Boulevard, Staten Island, NY 10314, USA; 3 Physics Department, College of the Holy Cross, 1 College Street, Worcester, MA 01610, USA

**Keywords:** ^19^F-NMR, FAAH, Anandamide carrier proteins, Human serum albumin

## Abstract

Fatty acid amide hydrolase (FAAH), the enzyme involved in the inactivation of the endocannabinoid anandamide (AEA), is being considered as a therapeutic target for analgesia and neuroprotection. We have developed a brain permeable FAAH inhibitor, AM5206, which has served as a valuable pharmacological tool to explore neuroprotective effects of this class of compounds. In the present work, we characterized the interactions of AM5206 with a representative AEA carrier protein, human serum albumin (HSA), using ^19^F nuclear magnetic resonance (NMR) spectroscopy. Our data showed that as a drug carrier, albumin can significantly enhance the solubility of AM5206 in aqueous environment. Through a series of titration and competitive binding experiments, we also identified that AM5206 primarily binds to two distinct sites within HSA. Our results may provide insight into the mechanism of HSA-AM5206 interactions. The findings should also help in the development of suitable formulations of the lipophilic AM5206 and its congeners for their effective delivery to specific target sites in the brain.

## Introduction

The endocannabinoid system has been implicated as a therapeutic target for analgesia, anti-emesis, and neuroprotection [1-3]. The blockade of fatty acid amide hydrolase (FAAH) can lead to a chronic elevation of the endocannabinoid anandamide (AEA) levels at the synapse [4,5]. Importantly, this produces few undesirable side effects typically associated with tetrahydrocannabinol (THC), the active psychotropic ingredient in marijuana [6]. For this reason, selective FAAH inhibition offers an attractive strategy to obtain the beneficial effects of CB1 receptor activation. Recently, it was reported that the cellular uptake of AEA can be significantly potentiated by a class of anandamide carrier proteins such as albumin and fatty acid binding proteins (FABPs) [7,8]. By inhibiting these anandamide carrier proteins, the uptake of AEA inside the cell was found to be drastically reduced. These findings provide a potential new therapeutic modality for neuroprotection through dual inhibition of FAAH and anandamide carrier proteins.

**Figure 1:**
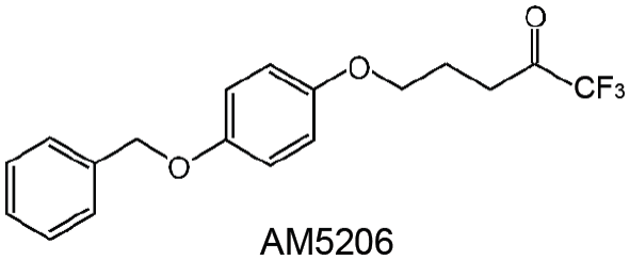
Structure of the trifluoromethyl ketone FAAH inhibitor AM5206.

In the course of developing potent and selective FAAH inhibitors, we have synthesized a series of trifluoromethyl ketone analogs [9,10]. One of the most successful compounds, AM5206 (1,1,1-trifluoro-5-[4-(benzyloxy)phenoxy]-2-pentanone, Figure 1), has served as a valuable pharmacological tool to explore the neuroprotective effects of FAAH inhibition [11-13]. It represents a new generation of reversible FAAH inhibitors that can cross the blood brain barrier and protect against neurodegenerative changes and cytoskeletal damages. To explore whether this class of FAAH inhibitors compete with AEA for binding to carrier proteins, we have studied the interactions of several trifluoromethyl ketone analogs with bovine serum albumin (BSA) [14]. We found that AM5206 has an affinity for serum albumin approximately one order of magnitude higher than its congeners. In the present communication, we reported a more detailed study of the interactions of AM5206 with human serum albumin (HSA) as a representative anandamide carrier protein.

Serum albumin has long been regarded as a carrier protein for various lipophilic endogenous and exogenous compounds to their specific targets [15,16]. As a drug carrier, albumin is a highly helical protein containing three homologous domains (I, II, and III), each consisting of two subdomains (A and B) [17-19]. Two structurally selective drug-binding sites, which are located in subdomains IIA and IIIA, are primarily associated with the delivery of various drug molecules [20-22]. A number of biophysical techniques have been used to study the interactions of ligands with albumin. These include fluorescence spectroscopy [23,24], gel chromatography [25], high-performance liquid chromatography (HPLC) [26], electrospray ionization mass spectrometry [27] and nuclear magnetic resonance (NMR). Although fluorinated natural products are relatively rare, it was reported that approximately 25% of drug molecules contain at least one fluorine atom [28]. For fluorine containing ligands, ^19^F-NMR has been of particular interest due to the fact that ^19^F has 100% natural abundance and ^19^F-signals are much more sensitive to changes in their chemical environment [29]. Additionally, because of the absence of fluorine atoms on proteins, the ^19^F-NMR spectra from mixtures of fluorinated ligands and their target proteins are much simplified.

In this work, we first employed ^19^F-NMR competition binding and titration experiments to study the site-specific binding preference of AM5206 with human serum albumin using warfarin, L-tryptophan and oleic acid [22,30,31] as site markers. We found that albumin can drastically enhance the solubility of the lipophilic AM5206 in an aqueous environment. We were also able to measure the exchange rates of AM5206 among these binding sites on albumin using two dimensional (2D) ^19^F-NMR exchange spectroscopy (EXSY) experiments [32]. The findings should help not only in elucidating the mechanism of HSA-AM5206 interactions, but also in developing suitable formulations for the effective delivery of AM5206 and its congeners to specific target sites in the brain.

## Materials and Methods

### Materials

Fatty acid free human serum albumin (HSA) (purity 96%), warfarin sodium, L-tryptophan, oleic acid, and 1 M HEPES buffer solution were purchased from Sigma-Aldrich (St. Louis, MO) and used without further purification. The FAAH inhibitor AM5206 (Figure 1) was synthesized in our laboratory and details of its synthesis will be published elsewhere. Deuterium oxide (D2O) and deuterated dimethyl sulfoxide (DMSO) were purchased from Cambridge Isotope Laboratories, Inc. (Cambridge, MA).

### Sample preparation

Human serum albumin (HSA) was dissolved in 10 mM HEPES buffer solution with 100 mM NaCl, 0.05% sodium azide (NaN_3_), and 5% D_2_O. AM5206 and the competing site markers warfarin, L-tryptophan and oleic acid were separately prepared in concentrated stock solutions in DMSO-d_6_ or D_2_O. For all NMR samples, AM5206 was first added to either the aqueous HEPES buffer or HSA solutions and the mixture was sonicated for 15 minutes in an ultrasonic water bath. In the competitive binding experiments, each of the competing site markers was added consecutively from their respective stock solutions.

### NMR measurement

All ^19^F-NMR experiments were carried out at 376.5 MHz using a Bruker AvanceII 400MHz NMR spectrometer using a 5 mm high-resolution BBFO probe. All spectra were recorded at 25°C using a 9.5-μs 90°-pulse and a 2-s repetition time without proton decoupling. Deuterated trifluoroacetic acid (CF_3_COOD) was used as a standard for chemical shift reference (-76.55 ppm). Two dimensional (2D) ^19^F-NMR exchange spectroscopy (EXSY) experiments were conducted using the NOESYPH pulse sequence with a series of mixing times from 0 to 100 ms. During the mixing time, signals can be transferred from one spin to another. The intensities of all diagonal and cross peaks were measured using the Bruker TOPSPIN software.

## Results and Discussion

### Site Selective Binding Characteristics of AM5206

**Figure 2:**
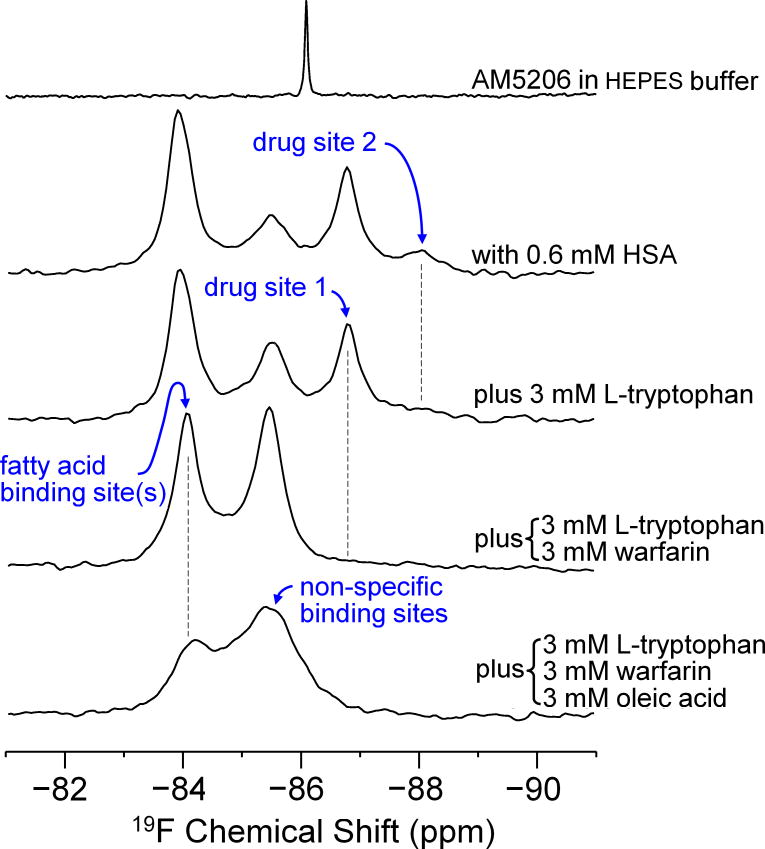
^19^F- NMR spectra of 0.6 mM AM5206, with 0.6 mM human serum albumin (HSA), and in the presence of one, two, or three of the competing site markers L-tryptophan, warfarin sodium and oleic acid.

The ^19^F-NMR spectrum of AM5206 in HEPES buffer consists of a single sharp peak at −86.18 ppm (Figure 2). However, when AM5206 was introduced into 0.6 mM HSA solution, three major peaks at −83.86, −85.44, and −86.77 ppm and a minor peak at −87.95 ppm were observed, indicating that there are multiple binding sites for AM5206 on HSA. In order to assign these peaks, we employed a series of competition binding experiments using three well characterized site markers, warfarin, L-tryptophan and oleic acid. Warfarin binds selectively to drug site 1 at subdomain *IIA*, while L-tryptophan is a typical ligand for drug site 2 at subdomain *IIIA* [22,30,31]. These two sites are the major drug binding sites on serum albumin. The naturally occurring ligand, oleic acid, can bind to a range of locations on serum albumin with three high affinity sites, one at subdomain *IB* and the other two at domain *III*. After adding 3 mM L-tryptophan, the minor peak at −87.95 ppm disappeared completely. This suggests that a very small fraction of AM5206 binds to drug site 2 on albumin and it can be displaced by L-tryptophan. In this same sample, when we added 3 mM warfarin, the signal at −86.77 ppm disappeared completely indicating that this peak arises from AM5206 binding to drug site 1 at subdomain *IIA*. With the addition of these two major drug site markers, the resonance at −85.44 ppm increased significantly. Upon further addition of 3 mM oleic acid, the signal at −83.86 ppm was partially reduced accompanied by a broadening and further increase of the peak at −85.44 ppm. We therefore assigned the –83.86 ppm peak to AM5206 binding at the fatty acid sites (FA sites) on albumin. The resonance at −85.44 ppm could be most probably attributed to non-specific binding of AM5206 to albumin.

**Figure 3:**
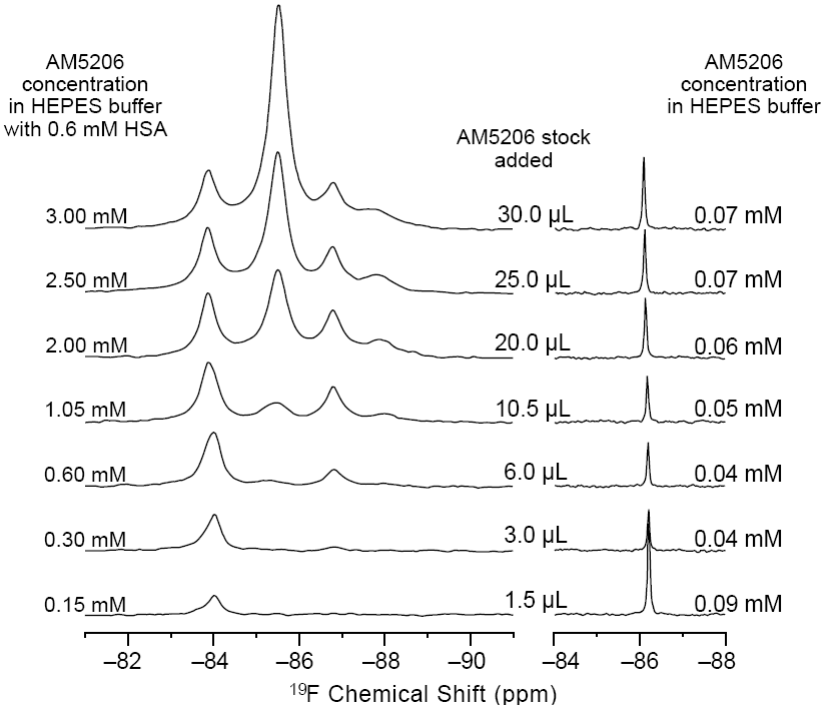
Left: ^19^F- NMR titration experiments of AM5206 into 0.6 mM HSA solution. Right: ^19^F-NMR spectra by adding the same amounts of AM5206 stock solution (50 mM in DMSO) into 500 μL HEPES buffer. The ligand concentrations listed in each spectrum reflect the soluble fraction of AM5206 as obtained by integrating the ^19^F-NMR signals (cf. Figure 4).

To further explore the binding characteristics of AM5206 with human serum albumin, we performed a series of titration experiments by adding AM5206 to 0.6 mM HSA solution. Figure 3 shows the ^19^F-NMR spectra with various HSA to AM5206 ratios. At HSA: AM5206 molar ratios of 1:0.25 and 1:0.5, only one single peak at −83.86 ppm was observed. This shows that the fatty acid sites (FA sites) on albumin are easily accessible by AM5206 at low concentrations. At a ratio of 1:1, a peak at −86.77 ppm started appearing. With further additions of AM5206, the resonances at −85.44 and −87.95 ppm also started to appear. At a HSA: AM5206 ratio of 1:5, the signal at −85.44 ppm is ~10× stronger than all other peaks, thus providing additional evidence that this −85.44 ppm peak is due to non-specific binding of AM5206 to HSA. In our earlier study of AM5206 with BSA, the ^19^F signal from AM5206 at drug site 1 and non-specific site coalesce due to fast exchange. Conversely, in our HSA experiments, the −86.77 ppm (drug site 1) and −85.44 ppm (non-specific binding) peaks remained separate over the entire course of our titration experiments, suggesting a tight binding of AM5206 to HSA compared to BSA [14]. Additionally, at all HSA:AM5206 ratios, no peak was observed at −86.18 ppm due to free AM5206 in solution, suggesting a fast exchange with the non-specific binding sites. Parallel NMR titration experiments were also conducted by adding the same amounts of AM5206 to HEPES buffer solution only. As shown in Figure 3, there was only one single sharp peak at −86.18 ppm due to free AM5206 in solution. The intensity of this peak remains virtually unchanged as the added amount of AM5206 stock increases from 1.5μL to 30 μL. No additional peak was observed due to any undissolved AM5206.

**Figure 4:**
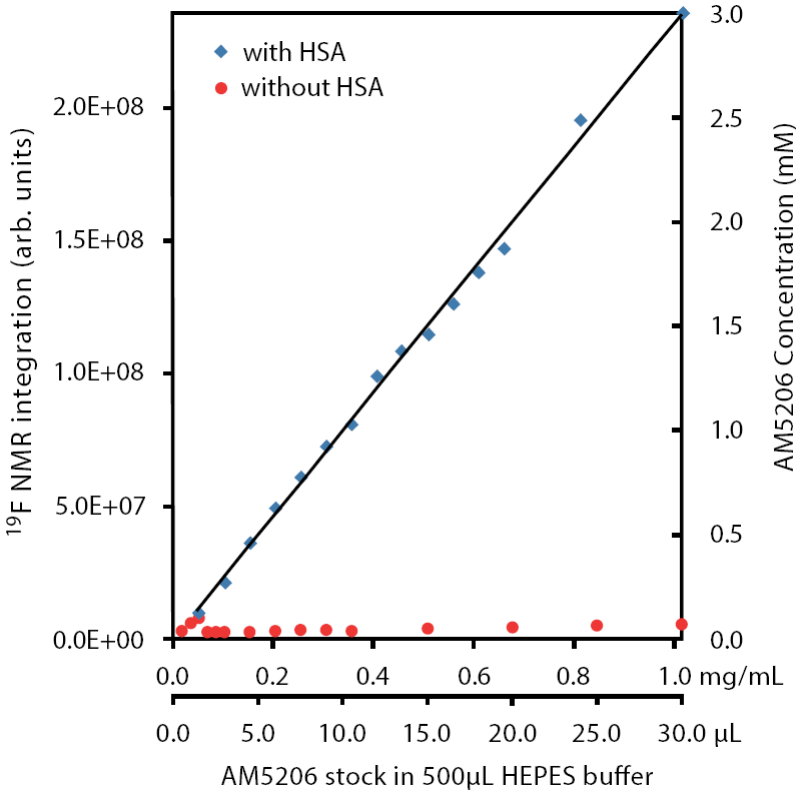
Integration of ^19^F-NMR signals from AM5206 in HEPES buffer with HSA (blue squares) and without HSA (red circles). The two horizontal axes represent the actual amount of AM5206 stock solution (50 mM in DMSO) added and the corresponding amount of AM5206 in the NMR sample (mg/mL). The vertical axis on the right shows the concentration of the soluble portion of AM5206.

It is interesting to compare the integrations of the ^19^F-NMR spectra from these two parallel titration experiments. Since NMR detects only the “soluble” fraction of AM5206 in all sample preparations, it presents a unique methodology to quantify its solubility [33-35]. To ensure accurate integration, we first measured the integrals of different ^19^F-NMR spectra recorded using a range of recycle delays from 1 s to 30 s and confirmed that a 2 s recycle delay between scans is adequate. In Figure 4, we plotted the total integral values against the amount of AM5206 added in each NMR sample. When titrated into the HSA solution, the integral values increased linearly with the addition of AM5206. The black line represents the best-fit of the data points according to the linear regression model, and the *r^2^* is found to be 0.993. Our data show that albumin can completely solubilize AM5206 up to 1.0 mg/ml concentration (30 μl AM5206 stock in 500 μl HSA solution). This corresponds to a 3.0 mM of AM5206 concentration in the HSA solution. In contrast, when AM5206 was added into HEPES buffer, the signal integral increased at the very beginning and leveled off for the remaining titration experiments. This corresponds to an AM5206 concentration of ~70 μM in the HEPES buffer solution. Therefore, albumin can significantly enhance the solubility of AM5206 in aqueous buffer solutions from ~70 μM to 3.0 mM, an approximately 50-fold increase.

### Exchange of AM5206 among different binding sites

**Figure 5:**
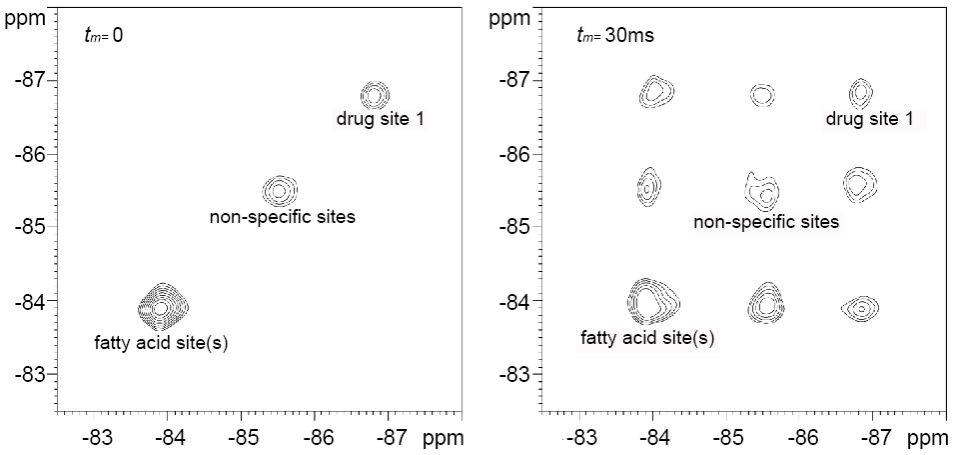
^19^F EXSY spectra of 1.2 mM AM5206 with 0.6 mM HSA in HEPES buffer. Left panel: mixing time t_*m*_ = 0. Right panel: t_*m*_ = 30 ms.

Spectral line shape analysis has been commonly used to study chemical exchange process. However, it is essentially limited to two-site exchange systems [36]. Two-dimensional exchange spectroscopy (EXSY) NMR experiments provide an excellent approach to investigate slow exchange process among different sites [37]. In order to determine the exchange rates for AM5206 among the different binding sites on HSA, 2D ^19^F-EXSY spectra were collected with mixing time *t_m_* ranging from 0 to 100 ms. (Figure 5) shows two representative EXSY spectra of AM5206 in HSA solution at a HSA: AM5206 molar ratio of 1:2. The minor signal at −87.95 ppm in 1D ^19^F-NMR spectrum was too weak to be observed in the 2D EXSY spectra. When *t_m_*=0 (no mixing), only three diagonal peaks were observed. As assigned earlier, peaks #1 (−83.86 ppm), #2 (−85.44 ppm), and #3 (−86.77 ppm) are due to AM5206 in fatty acid binding site(s), in the non-specific binding sites, and in drug site 1, respectively. When *t_m_* was gradually increased, six cross-peaks started to emerge and their intensities increased at the expense of the diagonal peak intensities, which is direct evidence for exchange among these three types of binding sites. We used the relative intensities of diagonal and cross peaks to quantitatively analyze the exchange rate constants among the three main sites identified in the 2D ^19^F-EXSY spectra [37,38]. The intensities of these nine peaks are given as functions of mixing time *t_m_*,

(1) $$I_{\mathrm{ij}}(t_{m})={\left(e^{-Rt_{m}}\right)}_{\mathrm{ij}}I_{\mathrm{ij}}(0),i=1,2,3\;\mathrm{and j}=1,2,3$$

where I_jj_ (0) are intensities of the three diagonal peaks at *t_m_*=0, R is a 3×3 matrix containing the pseudo first-order exchange rate constants k_ij_ and the spin-lattice relaxation time *T_1_*. Specifically,

(2) $$R=\left(\begin{array}{ccc} 1/T_{1}+k_{12}+k_{13} & -k_{21} & -k_{31}\\ -k_{12} & 1/T_{1}+k_{21}+k_{23} & -k_{32}\\ -k_{13} & -k_{23} & 1/T_{1}+k_{31}+k_{32} \end{array}\right)$$

It is not trivial to calculate the exponential of the **R** matrix since it is non-diagonal and asymmetric. It must be calculated by the following series expansion (we used 80 terms in the series), and R^n^ is the matrix multiplication of **R** by itself *n* times:

(3) $$e^{-Rt_{m}}=1\;-\;Rt_{m}+\frac{1}{2!}R^{2}t_{m}^{2}\;-\;\frac{1}{3!}R^{3}t_{m}^{3}+\ldots$$

To simplify calculations, we assumed that AM5206 at these three sites have the same *T_1_* value and used the fact that the pairs of exchange rate constants are related by relative populations as measured by *I_jj_*(0):

(4) $$k_{\mathrm{ij}}\;I_{\mathrm{ii}}\;(0)=k_{\mathrm{ji}}\;I_{\mathrm{jj}}\;(0)$$

**Figure 6:**
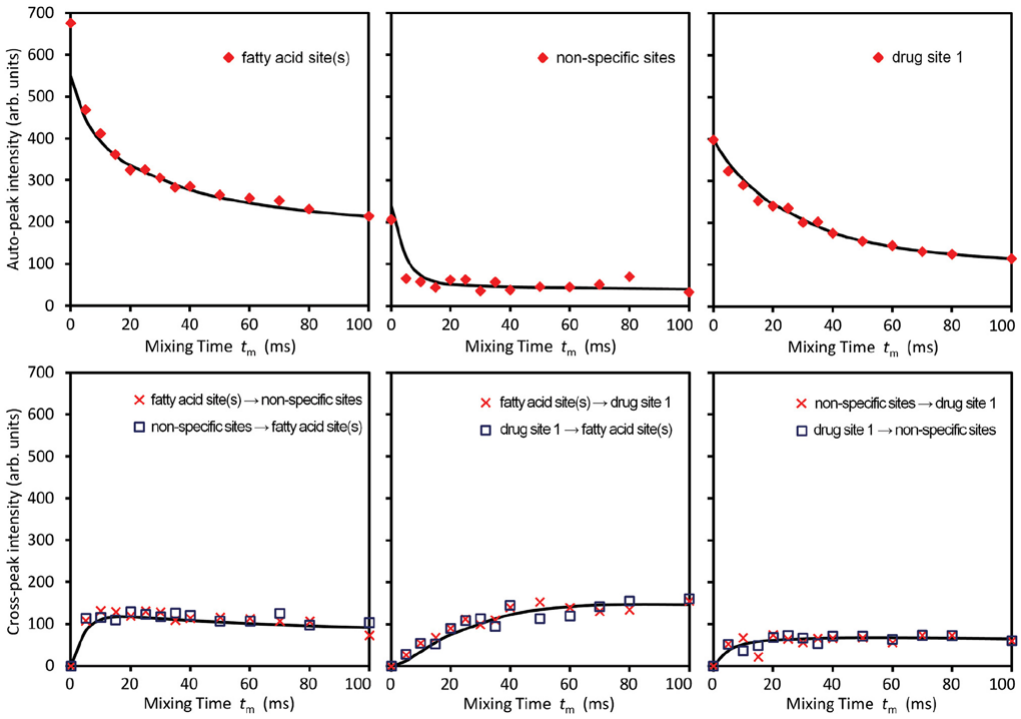
Different peak intensities as functions of mixing time *t_m_*. Top panels: three diagonal peaks. Bottom panels: three pairs of cross peaks. The solid lines represent calculated results using exchange rate constants *k_21_*=115 s^−1^, *k_13_*=1 s^−1^, *k_23_*=47 s^−1^, and spin-lattice relaxation time 0.5 s.

Therefore, there were only four adjustable parameters in **R**, namely, *k_21_, k_13_, k_23_*, and *T_1_*. Figure 6 shows simultaneous fits of the three diagonal peaks and the three pairs of cross peaks using the following parameters: *k_21_* = 115 s^−1^, *k_13_* = 1 s^−1^, *k_23_* = 47 s^−1^, and *T_1_* = 0.5 s.

Among these three diagonal peaks, we found that with the increase of *t_m_*, the −85.44 ppm (peak #2) exhibited a much faster decay than the other two diagonal peaks; consistent with our assignment that this peak represents the non-specific binding of AM5206. As for the cross peaks, we found that the 1-3 and 3-1 pair, which is due to exchange between fatty acid sites and drug site 1, has a much smaller initial slope than all the other cross-peaks. This observation clearly indicated that the exchange between these two specific binding sites is much slower than exchanges involving the non-specific sites. Our calculated exchange rate constants showed that *k_13_* is indeed almost two orders of magnitude smaller than either *k_21_* or *k_23_*.

**Figure 7:**
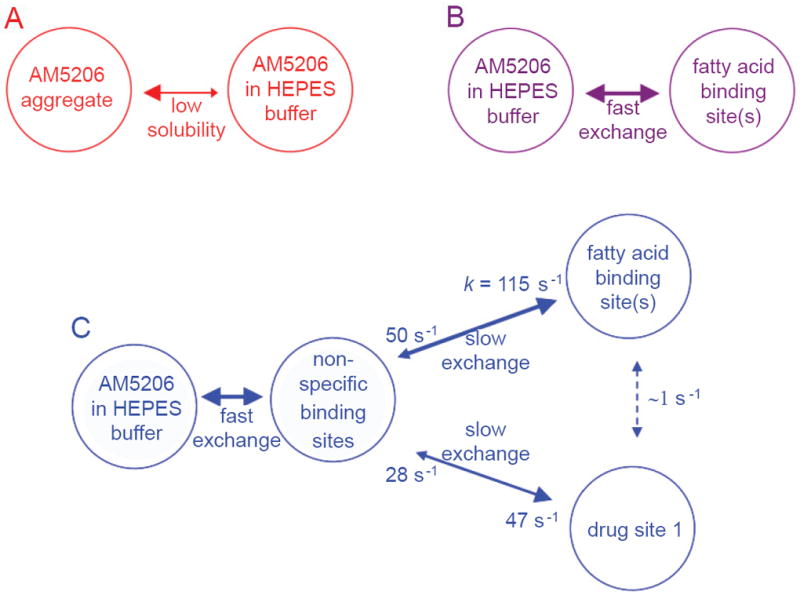
Proposed binding scenarios and the mechanism for the solubility enhancement by HSA: (A) In the aqueous buffer solution, AM5206 has a very low solubility (~70 μM), and most of it forms aggregates or even precipitates. (B) With HSA present, a fast exchange allows AM5206 to rapidly bind to the fatty acid binding site(s) and to dissolve the aggregated/precipitated AM5206. (C) More binding sites (namely, drug site 1 and non-specific binding sites) on HSA become available at higher concentrations of AM5206. The exchange rate constants among these binding sites are indicated in the diagram.

In the aqueous buffer solution, AM5206 has very low solubility (~70 μM) and it aggregates rapidly into various forms or even precipitates out of solution (Figure 7A). The addition of serum albumin can drastically increase the solubility of AM5206 and potentially dissolve all the aggregates. We now propose the following two different binding scenarios of AM5206 due to the multiple binding sites on human serum albumin. With low content of AM5206 (HSA:AM5206 ratio up to 1:0.5), it almost binds exclusively at the fatty acid binding site(s) on albumin (Figure 7B). This is probably because of the easy accessibility of some of the FA sites, particularly the FA site on subdomain *IB* which is the most open of all the presumed high-affinity pockets on HSA [18]. When higher amount of AM5206 is present in HSA solution, it starts to interact with many other different binding sites on albumin while AM5206 undergoes slow exchange among these binding sites (Figure 7C). Specifically, the exchange rates are 115 s^-1^ and 47s^-1^ for AM5206 to dissociate from the non-specific sites to the fatty acid site(s) or drug site 1, respectively. However, the exchange rate of AM5206 between the two specific binding sites (fatty acid site(s) and drug site 1) is ~1 s^-1^, almost two orders of magnitude smaller than those involving the non-specific binding sites. This observed spectroscopic exchange can be explained through a direct transfer of AM5206 between fatty acid site(s) and drug site 1. This very slow process is also compatible with an alternative explanation according to which AM5206 undergoes exchanges indirectly through the intermediacy of the non-specific sites. As for AM5206 at the non-specific sites, there exists a fast exchange with the free AM5206 present in the HSA solution. The more AM5206 added into the HSA solution, the more it associates directly to the non-specific binding sites. In the present study, the non-specific binding sites are not saturable over the entire titration experiments with HSA:AM5206 ratios up to 1:5. The complexity of the binding scenarios of serum albumin, in conjunction with the poor aqueous solubility of ligands, underscores the difficulty of obtaining accurate dissociation constants for each of the binding sites. This helps explain the frequent discrepancies of reported affinity values of drug molecules with albumin in the literature.

## Conclusions

The interactions between a selective FAAH inhibitor AM5206 and human serum albumin have been studied using ^19^F NMR technique. The primary binding sites for AM5206 are drug site 1 located at subdomain *IIA* as well as fatty acid site(s) at subdomain *IB* and domain *III*. With excess amount of AM5206 added, it tends to associate with many of the non-specific binding sites on albumin. This drastically increases the aqueous solubility of AM5206. To further understand the binding events, we have also determined the exchange rates of AM5206 among various binding sites on HSA. Comparing to our earlier results with BSA, AM5206 has a much tighter binding to drug site 1 on human serum albumin. It has been demonstrated that the endocannabinoid anandamide also binds to HSA. It can thus be argued that AM5206 may compete with anandamide for HSA binding. The result of such a potential competition would be the enhancement of free anandamide levels. We conclude that in addition to its role as a FAAH inhibitor, AM5206 may further enhance its effects through the release of anandamide from HSA. We are now in the process of identifying the specific sites for anandamide binding with HSA. Further experiments are also underway to examine whether AM5206 can compete effectively with anandamide for binding with albumin.

## Abbreviations

AEA: N-arachidonoylethanolamine, anandamide
FAAH: Fatty Acid Amide Hydrolase
HSA: Human Serum Albumin
